# Microbial community structure in tinea corporis and antifungal activity of *Lactiplantibacillus plantarum* YM-4–3 strain

**DOI:** 10.3389/fcimb.2026.1839198

**Published:** 2026-08-24

**Authors:** En Yang, Ningnan Zhang, Zhen Zhang, Mengyue Li, Chenjian Liu, Xiaoran Li

**Affiliations:** 1 Faculty of Life Science and Technology, Kunming University of Science and Technology, Kunming, China; 2 The First People’s Hospital of Yunnan Province, Kunming, China

**Keywords:** antimicrobial activity, high-throughput sequencing, *lactiplantibacillus plantarum*, skin microbiota, tinea corporis

## Abstract

**Objective:**

Tinea corporis is a common superficial fungal infection associated with skin microbiota dysbiosis. This study aimed to investigate the microbial community structure in lesional and peri-lesional skin of tinea corporis patients and to evaluate the antifungal activity of a probiotic *Lactiplantibacillus plantarum* strain against dermatophytes and *Candida albicans*.

**Methods:**

Fifty skin-scraping samples were collected from 25 tinea corporis patients (paired lesional and adjacent non-lesional samples). Bacterial and fungal communities were analyzed by high-throughput sequencing of 16S rDNA (V3–V4) and internal transcribed spacer (ITS) regions, respectively. An antifungal *L. plantarum* YM-4-3 strain was isolated, and its cell-free supernatant was assessed for antifungal activity, stability, and minimum inhibitory concentrations against *Trichophyton rubrum* and *C. albicans*. Scanning electron microscopy, transmission electron microscopy, and quantitative PCR were employed to investigate ultrastructural damage and bacteriocin-related gene expression.

**Results:**

At the phylum level, Proteobacteria (64.6%), Actinobacteria (18.4%), and Firmicutes (15.2%) dominated bacterial communities, whereas Basidiomycota (57.4%) and Ascomycota (42.3%) accounted for 99.7% of fungal reads. Genus-level profiling revealed that *Pseudomonas*, *Corynebacterium*, and *Staphylococcus* were abundant at both sites, yet *Lactobacillus* was significantly enriched adjacent to lesions (2.6% vs. 0.6%). Among fungi, *Cryptococcus* and *Trichophyton* prevailed, with *C. albicans* more abundant in lesions. The *L. plantarum* YM-4-3 cell-free supernatant exhibited the strongest inhibition against *C. albicans* when grown in MRS with 2.0% NaCl, reaching pH 4.06 after 12 h co-culture. SEM showed the supernatant disrupted the cell wall and membrane of *T. rubrum*. qPCR indicated a 7.5-fold up-regulation of the bacteriocin gene plnJK under 2.0% NaCl. Ketoconazole (MIC < 1 μg/mL) and terbinafine (MIC 1–2 μg/mL) were highly active against T. rubrum, while amphotericin B (MIC < 1 μg/mL) was most effective against C. albicans. Although the concentrated L. plantarum YM-4-3 supernatant had higher MICs (64–128 mg/mL), inhibition rates exceeded 87%, declining with increased salinity.

**Conclusions:**

These findings reveal distinct microbial signatures associated with tinea corporis and demonstrate the salt-dependent antifungal potential of *L. plantarum* YM-4-3. This study provides a theoretical basis for novel antifungal probiotic formulations and offers a new strategy to circumvent adverse effects and resistance associated with conventional antifungal therapies.

## 1 Introduction

Tinea corporis, also known as ‘ringworm,’ is a common superficial fungal infection (Gupta et al., 2016). Approximately 40 fungal species can cause tinea corporis (Haque et al., 2020), The main causative fungi of tinea corporis belong to the genera *Microsporum*, *Trichophyton*, *Epidermophyton*, *Nannizzia*, *Paraphyton*, *Lophophyton*, and *Arthroderma* (Nweze and Eke, 2016; Liang et al., 2026). Some *Malassezia* species may also cause superficial infections. Global incidence of fungal skin infections shows a persistent upward trend (Alehashemi et al., 2025; Chua et al., 2025). According to the World Health Organization (WHO), approximately one-third of the global population is affected by fungal infections, and 90% of individuals experience at least one such infection during their lifetime (Aimoldina et al., 2025). In China, pathogenic fungal infections represent an escalating public health priority, with expanding high-risk demographics and rising antifungal resistance posing concurrent challenges across clinical and environmental settings (Miao et al., 2026). *T. rubrum* and *C. albicans* are two common pathogenic fungi capable of causing superficial fungal infections in humans. Global warming has further reshaped fungal epidemiology by enhancing thermal tolerance, enabling pathogens to overcome mammalian thermal defenses and thereby increasing virulence and antifungal resistance (Jiang et al., 2026). Among these, *T. rubrum* is the most prevalent dermatophyte and a major causative agent of superficial mycoses, accounting for approximately 90% of chronic dermatophytosis cases (Rezaie et al., 2000). In recent years, the misuse of antibiotics and antifungal drugs has led to the emergence of drug-resistant strains, posing a serious challenge to clinical treatment. A study by Cañete-Gibas et al., 2023 revealed that 18.6% of 271 dermatophyte isolates exhibited resistance to terbinafine (MIC ≥0.5 µg/mL), including 21 *T. rubrum* and 21 *T. indotineae* strains. In China, uncommon and drug-resistant dermatophytoses frequently lack standardized diagnostic and therapeutic protocols and remain refractory to conventional antifungal therapies (Liang et al., 2026). This finding has prompted the exploration of alternative treatment strategies.

Natural antimicrobial substances (e.g., plant extracts, bioactive compounds, and essential oils) have shown significant antifungal activity. According to Qamar et al. (2020) it was found that green synthesized copper oxide nanorods (CuO NRs) from the aqueous extract of Momordica charantia showed significant inhibition against *T. rubrum*. Three phenolic compounds extracted from pickled radish showed different inhibitory effects on *C. albicans*. Among them, 2,6-dihydroxyacetophenone (DHAP) and 4-hydroxybenzaldehyde (HBA) showed significant antimicrobial activity, whereas 4-hydroxyphenethyl alcohol (4-HPEA) showed a weak inhibitory effect (Li et al., 2020). *Eucalyptus* spp. essential oils not only effectively suppress the growth of phytopathogenic and wood-decaying fungi, but also exhibits inhibitory activity against human pathogenic fungi such as *C. albicans*. The antifungal mechanism may be attributed to terpenoid compounds in the essential oil disrupting fungal cell membrane integrity and interfering with normal physiological processes (Yip et al., 2024).

Moreover, probiotic metabolites demonstrated significant antimicrobial activity. Studies have revealed that *L. paracasei* strain 28.4 inhibits *C. albicans* biofilm formation and hyphal development either through direct contact or via its metabolites. This strain downregulates the expression of biofilm-associated genes in *C. albicans* (e.g., *ALS3*, *HWP1*, *EFG1*, and *CPH1*), thereby attenuating its pathogenicity (Rossoni et al., 2018). *L. rhamnosus* protects oral epithelium from *C. albicans* infection by competitively occupying epithelial adhesion sites, thereby reducing fungal binding capacity and preventing adherence, invasion, and subsequent tissue damage (Naglik et al., 2017). Additionally, lactobacilli secrete antimicrobial compounds, including organic acids, short- chain fatty acids, cyclic dipeptides, and bacteriocins (Schnürer and Magnusson, 2005; Reis et al., 2012). These secondary metabolites establish a chemical barrier that effectively prevents pathogenic microorganisms from colonizing and invading. Lactobacilli represent promising candidates for developing novel clinical bactericidal agents (Yang et al., 2015).

Therefore, this study aimed to investigate the microbial community structure and successional patterns in both lesional and peri-lesional skin of tinea corporis patients. Using *T. rubrum* and *C. albicans* as indicator strains, we identified probiotic strains with optimal antifungal activity. The findings may provide novel theoretical foundations for dermatological diagnosis and treatment while offering innovative strategies to address adverse effects and drug resistance associated with conventional antifungal therapies.

## 2 Materials and methods

### 2.1 Materials

This study enrolled patients diagnosed with tinea corporis from the First People’s Hospital of Yunnan Province. Skin scrapings were collected aseptically from both lesional sites and adjacent non-lesional areas (3 cm from lesions) using sterile scalpels. A total of 50 samples were obtained from 25 patients (paired lesional/peri-lesional samples per patient). All specimens were immediately placed in portable cooling containers, transported to the laboratory, and stored at -20°C until further processing.

### 2.2 Antifungal lactic acid bacteria screening under salt stress

Twenty-five LAB strains previously isolated by our laboratory from traditional fermented foods in Yunnan Province were used as experimental strains, with *T. rubrum* (kindly provided by the Department of Dermatology, Shanghai Changzheng Hospital) serving as the indicator fungus. Initial screening for antifungal activity was performed using the agar-spot-on-lawn method (Omar et al., 2006). Briefly, each LAB strain was inoculated (4% v/v) into 5 mL of MRS broth and incubated statically at 37°C for 18 h. The cultures were then vortexed, and 10 µL aliquots were spotted onto MRS agar plates containing NaCl gradients (0%, 0.9%, 2%, and 3%). After 24 h of incubation at 37°C to allow colony formation, the plates were overlaid with PDA medium inoculated with *T. rubrum* (final concentration 1×10^4^ CFU/mL). The co-culture systems were incubated at 37°C for 48 h, after which the antifungal activity was assessed by measuring inhibition zones.

### 2.3 Effects of NaCl, KCl, and Sorbitol on acid production by *L. plantarum* YM-4–3 fermentation

Fifty milliliters of MRS broth medium were prepared with varying concentrations (0.0%, 0.9%, 2.0%, and 3.0%) of sodium chloride (NaCl), potassium chloride (KCl), and sorbitol. The activated *L. plantarum* YM-4–3 strain was inoculated into the prepared media at a concentration of 1×10^6^ CFU/mL and incubated at 37°C. The initial pH was adjusted to 7.0. Samples (2 mL) were collected at 12 h, 36 h, 60 h, and 84 h to measure and record pH values.

To evaluate thermal stability, the cell-free supernatant of the *L. plantarum* YM-4–3 strain was exposed to varying temperatures (65°C, 80°C, and 100°C) for 1 h. pH stability was assessed by adjusting the supernatant pH to 6.5. Enzymatic effects were examined by treating samples with proteinase K and catalase (both at 1 mg/mL final concentration), followed by incubation at 45°C for 3 h and subsequent heat inactivation at 65°C for 30 min.

### 2.4 Stability evaluation

The antifungal activity against *C. albicans* was assessed using a microdilution method in 96-well plates. Each well contained a total volume of 150 µL, consisting of 130 µL of treated or untreated *L. plantarum* YM-4–3 strain fermentation supernatant and 20 µL of fungal spore suspension (1.0×10^4^ CFU/mL). The plates were incubated at 28°C for 48 h and 72 h, followed by optical density measurement at 530 nm using a microplate reader. Appropriate controls were included, with the MRS medium serving as the blank control. All experiments were performed in triplicate.

### 2.5 Determination of the MIC

The MIC of antifungal agents was determined using a 96-well broth microdilution method following CLSI reference methods for yeasts (*C. albicans*) and filamentous fungi (*T. rubrum*), with minor modifications. Each well contained a final volume of 200 µL, with wells 1–10 receiving 100 µL of serially diluted drug solutions (concentration gradients: 0.03-16 µg/mL for amphotericin B, ketoconazole, itraconazole, and terbinafine; 0.125-128 µg/mL for fluconazole and flucytosine; 0.25–128 mg/mL for concentrated fermentation supernatant) plus 100 µL of fungal suspension (final concentration 1×10^4^ CFU/mL). Well 11 served as the positive control (100 µL fungal suspension + 100 µL RPMI 1640 medium), whereas well 12 was the negative control (200 µL RPMI 1640 medium). The 96-well plates were incubated statically at 28°C for 72 h, after which a microplate reader was used to measure the optical density at 530 nm. Three independent experiments were performed for each experimental group.

### 2.6 DNA extraction and bioinformatics analysis of the skin microbiome patients with tinea corporis based on Illumina high-throughput sequencing

The collected skin scrapings were homogenized in a sterile mortar with 500 µL of lysis buffer, followed by microbial genomic DNA extraction according to Schmidt et al. (1991). For target gene amplification, 515F (5′-GTGCCAGCMGCCGCGGTAA-3′) and 909R (5′-TTTCAGYCTTGCGRCCGTAC-3′) universal primers targeting the V3-V4 region of bacterial 16S rDNA were used, while fungal ITS regions were amplified using ITS1F (5’-CTTGGTCATTTAGAGGAAGTAA-3’) and ITS2R (5’-GCTGCGTTCTTCATCGATGC-3’). PCR amplification was performed for 30 cycles under the following conditions: initial denaturation at 95°C for 5 min; 30 cycles of 95°C for 30 s, 55°C for 1 min, and 72°C for 30 s; followed by a final extension at 72°C for 10 min. To reduce heteroduplex formation, PCR products were diluted 10-fold and re-amplified for 5 additional cycles under identical conditions. Amplification success was verified by 1% (w/v) agarose gel electrophoresis.

Paired-end sequencing was conducted on the Illumina MiSeq platform, with barcode-based sample demultiplexing. Raw sequences were quality-filtered by removing primer sequences and low-quality reads, and bacterial and fungal sequences shorter than 400 bp and 200 bp discarded, respectively. Bacterial 16S rDNA sequences were taxonomically classified against the SILVA database (SSU rRNA V102, Release 119) using the classify. seqs command in mothur (v1.41.20) while fungal ITS sequences were annotated using the UNITE database (Release 7.0) with the RDP classifier Bayesian algorithm at 97% similarity threshold. Operational taxonomic units (OTUs) were clustered at 97% sequence similarity for alpha diversity analysis, with ACE and Chao1 indices calculated at 0.03 cutoff and rarefaction curves generated.

### 2.7 Morphological identification of *T. rubrum* strains

After incubating *T. rubrum* on various media at 28°C for 5 days, we recorded the colony characteristics (morphology, color, and growth rate).

#### 2.7.1 Colony diameter measurement

The experimental strains were initially cultured on Sabouraud Dextrose Agar (SDA) at 28°C for 7 days. Aseptically, a sterile 9 mm diameter punch was used to cut mycelial plugs from the edge of the colony, and then transfer them to SDA, yeast extract peptone dextrose (YEPD), and potato dextrose agar (PDA). All cultures were incubated in parallel at 28°C. The colony diameters were measured daily for 10 days, and the mean values were calculated for statistical analysis.

#### 2.7.2 Mycelial growth detection and plotting of growth curves

The mycelia of the same age were cut with a perforator (9 mm in diameter) and inoculated into SDA, YEPD, and PDB liquid media. The cultures were first incubated statically at 28°C for 48 h and then transferred to a shaker (28°C, 250 rpm) for 14 days of shaking cultivation. Starting from the 4th day, six vials of culture were randomly taken each day, centrifuged at 4°C for 10 min at 8000 rpm, and washed twice with PBS buffer. The mycelium was then collected and dried at 80°C until constant weight, and the dry weight was determined. The growth curves of *T. rubrum* in different culture media were plotted based on these measurements.

### 2.8 Effect of *L. plantarum* YM-4–3 fermentation supernatant on cell membranes of *T. rubrum*

The *L. plantarum* YM-4–3 strain was cultured in MRS broth at 37°C with agitation (180 rpm) for 72 h. Cell-free supernatant (1 × MIC) was collected, and 1 mL was inoculated into PDB containing *T. rubrum* at an initial concentration of 1×10^4^ CFU/mL. Both experimental and control groups were incubated at 28°C with shaking (180 rpm) for 96 h, with the negative control receiving sterile physiological saline (0.85% NaCl) instead of supernatant. Post-culture, the samples were centrifuged (8,000 r/min, 10 min), and the pellets were washed twice with 0.85% saline, fixed overnight in 2.5% glutaraldehyde (1.5 mL), and rinsed with PBS. Subsequent dehydration was performed using an ethanol gradient, followed by air-drying at room temperature. To assess morphological alterations in T. rubrum, the prepared samples were analyzed by scanning electron microscopy (SEM) and transmission electron microscopy (TEM).

### 2.9 Inhibitory effects of *L. plantarum* YM-4–3 bacteriocin on *C. albicans* growth and analysis of related gene expression

For monoculture controls, *C. albicans* was inoculated into SDB at 1×10^4^ CFU/mL and cultured at 35°C. For co-culture experiments, *L. plantarum* YM-4–3 strain and *C. albicans* were simultaneously inoculated into SDB at 1×10^6^ CFU/mL and 1×10^4^ CFU/mL, respectively, followed by incubation at 35°C. Samples (2 mL) were collected at 4 h intervals over 48 h for quantification of viable counts (CFU enumeration) and pH measurement.

Total RNA was extracted using Sangon Biotech kits, followed by cDNA synthesis with HiScript II Q RT SuperMix for qPCR (Vazyme). PCR amplification was performed targeting five selected genes (primer sequences listed in **Table 1**), using 16S rRNA and *fus*A_2_ as internal reference genes. Relative gene expression levels were quantified via RT-qPCR and calculated using the 2^−ΔΔCT^ method.

**Table 1:** Primers for fluorescent real-time quantitative PCR.

Gene	Sequence (5’ to 3’ direction)
16S rRNA	AACCCAACATCTCACGACAC
	GCAAGGCTGAAACTCAAAGG
*fus*A2	CCCATGATGGTGCTTCACAA
	TCGTGGCAGCAGAGGTAATG
*pln*C	GGCGACAGGAGATTTACAAGAG
	CAAGGTAACTGCTGCCGAAT
*pln*IEF	GAGAACCTGACACAATACTGACC
	CGCTTCGATGATGACTGCTT
*pln*RL	GCGTGAAGGCTATCCATACTCTT
	TGTTCTGGAAGTCACTGCGATT
*pln*JK	CTGCCAGCTTCGCCATCATA
	AGTTGAACGGGGTTGTTGGG
*pln*GHSTUVW	CCCGCAGGAACCATACATCTTT
	GTTGTCGCTGACCACCTGATAG

### 2.10 Statistical analysis

All experiments were performed in triplicate, the statistical analysis was conducted using GraphPad Prism 10, employing one-way analysis of variance or Student’s t-test. All data are presented as mean ± standard deviation.

## 3 Results

### 3.1 Sample information

The male-to-female ratio among the 25 randomly enrolled patients was approximately 1:1 (11 males and 14 females). Participant ages ranged from 11 to 45 years, with the majority (84%) aged 18–45 years. The disease duration varied from 30 days to 10 years, with 72% of cases exceeding 1 year. The most prevalent superficial fungal infection was tinea pedis (80%), followed by tinea cruris (16%) and tinea manuum (4%). Detailed clinical profiles are presented in **Table 2**.

**Table 2:** Study subjects’ information table.

Patient group	Age (years)	Sex	Affected site	Number of patients (n)	Disease duration (years)
Adolescent Group	11–17	Male	–	0	–
		Female	Foot	2	0.1–0.25
Young Adult Group	18–45	Male	Hip	4	0.25–2
			Foot	7	1.0–10
		Female	Foot	11	0.2–4
			Hand	1	2

### 3.2 Results of strain screening

**Figure 1:**
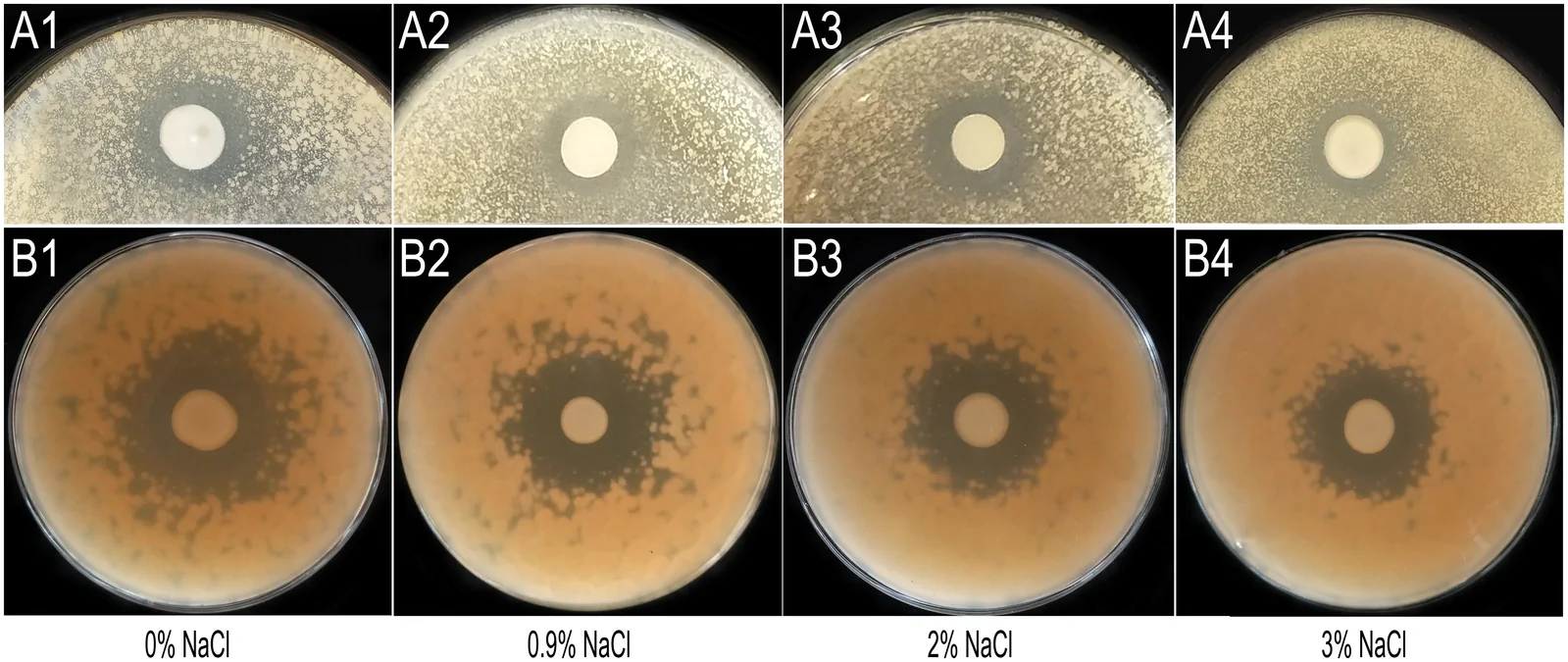
The antimicrobial effects of the *L. plantarum* YM-4–3 strain on *C. albicans* and *T. rubrum* under different salt concentrations are shown. **(A)** illustrates the inhibitory effects of the *L. plantarum* YM-4–3 strain on *C. albicans* at various salt concentrations. **(B)** depicts the inhibitory effects of the *L. plantarum* YM-4–3 strain on *T. rubrum* under the same conditions.

Preliminary and secondary screening of 25 LAB strains against *T. rubrum* and *C. albicans* revealed that the *L. plantarum* YM-4–3 strain exhibited the most potent inhibitory effects against both pathogenic fungi (**Figure 1**). Bacteriostatic activity showed a significant inverse correlation with NaCl concentration, demonstrating enhanced efficacy under salt-free conditions.

### 3.3 Acid production by *L. plantarum* YM-4–3 strain in response to NaCl, KCl, and sorbitol

As shown in **Table 3**, *the L. plantarum* YM-4–3 strain exhibited consistent pH reduction patterns in standard MRS medium (without NaCl, KCl, or sorbitol), with pH values decreasing significantly to approximately 4.22 by 36 h and stabilizing thereafter. The addition of sorbitol (0.0%–3.0%) had no significant effect on pH, indicating negligible interference with acidogenic metabolism. In contrast, both NaCl and KCl caused transient pH elevation at 12 h. However, during subsequent acidification, NaCl exerted stronger inhibitory effects on pH modulation. Notably, at 2.0%–3.0% concentrations, NaCl-treated cultures achieved significantly lower final pH values (4.06–4.10 at 36 h) than KCl-supplemented groups.

**Table 3:** Changes of pH value of the *L. plantarum* YM-4–3 strain after adding different concentrations of substances.

Substance	Concentration	Time			
		12h	36h	60h	84h
NaCl	0%	6.23	4.22	4.23	4.23
	0.9%	6.43	4.14	4.13	4.15
	2.0%	6.41	4.06	4.06	4.10
	3.0%	6.42	4.19	4.04	4.07
KCl	0%	6.21	4.22	4.23	4.24
	0.9%	6.56	4.20	4.20	4.20
	2.0%	6.56	4.20	4.17	4.17
	3.0%	6.56	4.22	4.15	4.15
Sorbitol	0%	6.20	4.22	4.22	4.22
	0.9%	6.51	4.21	4.22	4.22
	2.0%	6.50	4.20	4.22	4.22
	3.0%	6.52	4.21	4.22	4.21

### 3.4 Stability of the fermentation supernatant from *the L. plantarum* YM-4–3 strain

**Figure 2:**
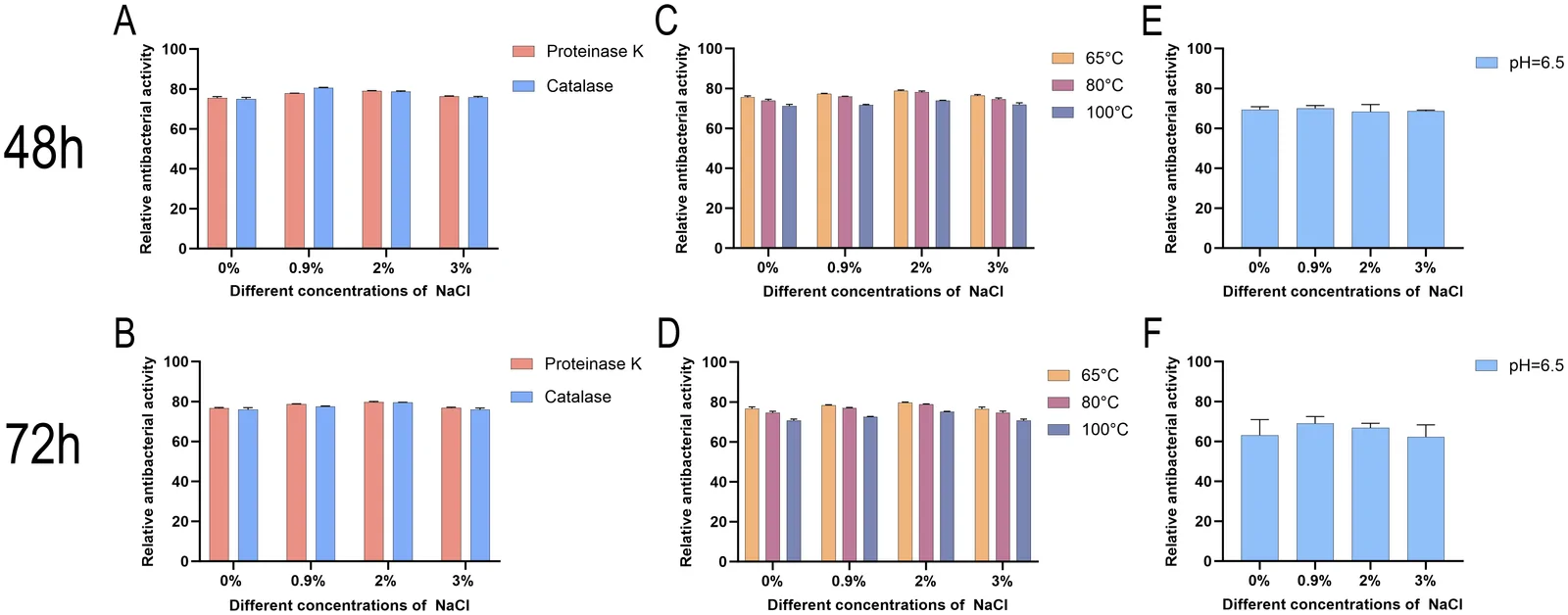
The stability of the *L. plantarum* YM-4–3 strain fermented supernatant under different salt treatments was evaluated at 48 h and 72 h. **(A, B)** show enzyme stability. **(C, D)** display thermal stability. Figures **(E, F)** demonstrate pH stability.

As illustrated in **Figures 2A, B**, proteinase K treatment markedly reduced the antifungal activity of the *L. plantarum* YM-4–3 strain supernatant against *C. albicans* across all NaCl concentrations (0-3%), confirming protease-sensitive protein components. Catalase treatment caused only a minor reduction in activity, suggesting limited peroxide-mediated effects. Temperature sensitivity tests (**Figures 2C, D**) demonstrated a progressive loss of activity with increasing heat (65-100°C), indicating thermolabile active compounds. Adjusting the pH from 4.5 to 6.5 (**Figures 2E, F**) significantly decreased inhibition, revealing pH-dependent efficacy. Collectively, the antifungal activity of the *L. plantarum* YM-4–3 strain primarily derives from heat- and protease-sensitive proteins.

### 3.5 MIC

As presented in **Table 4**, the six tested antifungals showed markedly different efficacy against *T. rubrum* and *C. albicans*. *T. rubrum* demonstrated highest sensitivity to ketoconazole (MIC<1 ug/mL) and terbinafine (1 ≤ MIC ≤ 2 ug/mL), but was resistant to 5-fluorocytosine (MIC≥64 ug/mL). *C. albicans* exhibited optimal susceptibility to amphotericin B (MIC<1 ug/mL) with resistance to ketoconazole (MIC>32 ug/mL), fluconazole (MIC≥64 ug/mL), terbinafine (MIC≥64 ug/mL) and 5-fluorocytosine (MIC≥128 ug/mL). *L. plantarum* YM-4–3 strain concentrated supernatant displayed NaCl concentration-dependent activity: MICs against *T. rubrum* increased from <64 mg/mL (0% NaCl) to ≥64 mg/mL (0.9% NaCl) and 64–128 mg/mL (2-3% NaCl), while MICs for *C. albicans* remained at 64 mg/mL (0-0.9% NaCl) before increasing to <128 mg/mL at higher salt concentrations (2-3% NaCl).

**Table 4:** Antimicrobial activity of various substances against *T. rubrum* and *C. albicans.*.

*A T. rubrum*			
Antifungal agents	MIC(µg/mL)	Fermentation supernatant	MIC(mg/mL)
5-Fluorocytosine	≥64	0.0% NaCl	<64
Amphotericin B	≤8	0.9% NaCl	≥64
Terbinafine	1–2	2.0% NaCl	64–128
Fluconazole	16–32	3.0% NaCl	64–128
Itraconazole	≤4		
Ketoconazole	<1		
*B C. albicans*			
5-Fluorocytosine	≥128	0.0%NaCl	≤64
Amphotericin B	<1	0.9%NaCl	>64
Terbinafine	≥64	2.0%NaCl	<128
Fluconazole	≥64	3.0%NaCl	<128
Itraconazole	>32		
Ketoconazole	<1		

### 3.6 Principal component analysis of the dermatophyte microbiota community structure

**Figure 3:**
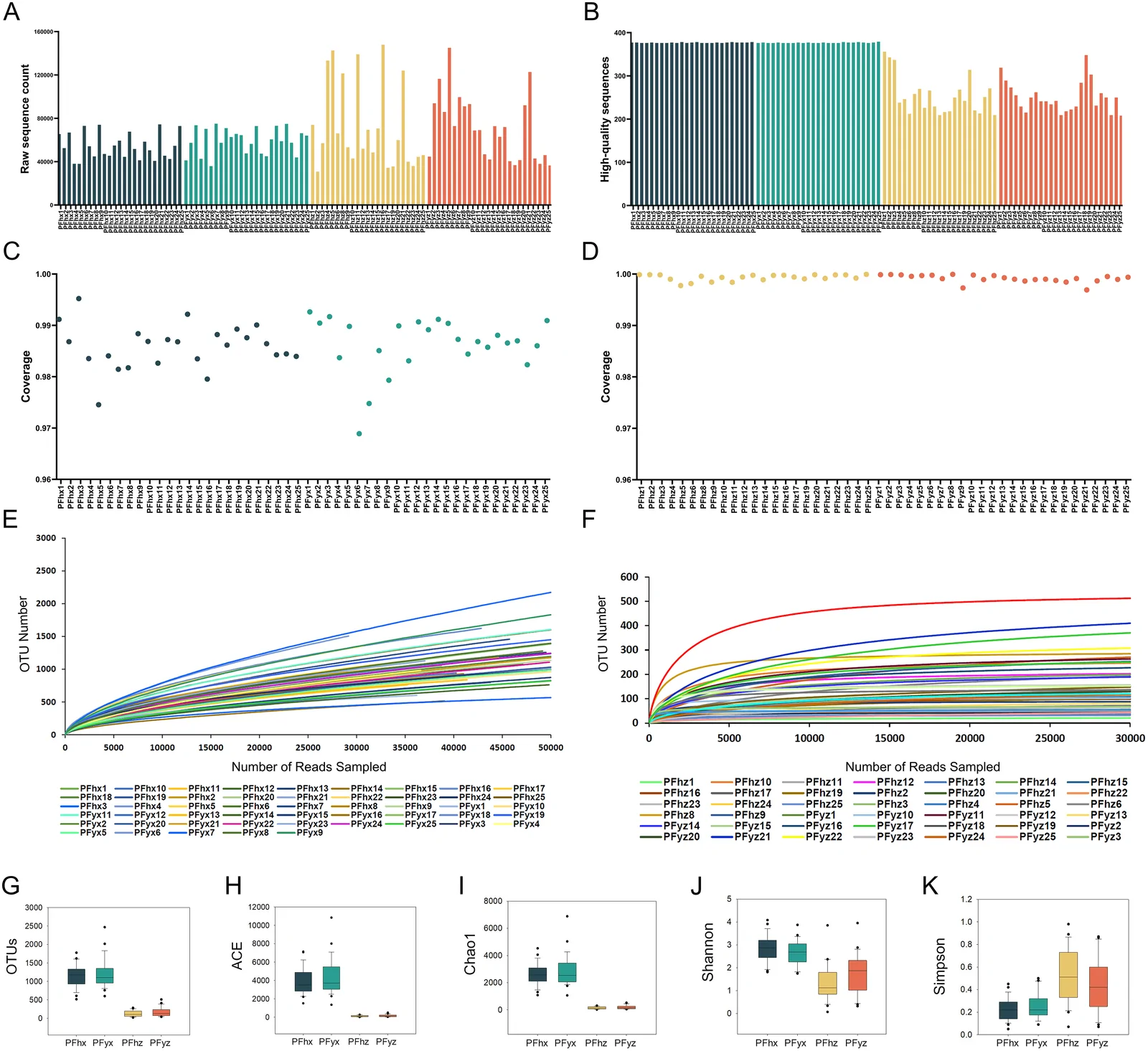
Analysis of the microbial community in tinea corporis samples: Alpha diversity assessment based on OTUs and Beta diversity comparison driven by Rarefaction curves. **(A)** shows the unprocessed paired-end sequences of 16S and ITS. **(B)** displays the high-quality sequences of 16S and ITS. **(C)** is the Coverage curve for bacteria. **(D)** is the Coverage curve for fungi. **(E)** is the Rarefaction curve for bacteria. **(F)** is the Rarefaction curve for fungi. **(G–K)** represent the OTU counts, ACE index, Chao1 index, Shannon index, and Simpson index of the tinea corporis microbiota, respectively. PFhx indicates bacteria from the skin lesion area, PFyx indicates bacteria from the surrounding skin area, PFhz indicates fungi from the skin lesion area, and PFyz indicates fungi from the surrounding skin area.

High-throughput sequencing of the 16S rDNA V3-V4 and ITS regions generated 2,841,652 and 3,441,724 raw paired-end reads, respectively (**Figure 3A**). After quality control, 2,610,072 high-quality 16S sequences (mean length >370 bp) and 2,610,072 ITS sequences (mean length >230 bp) were retained (**Figure 3B**). Rarefaction analysis indicated sufficient sequencing depth for bacterial communities (coverage >97%), though curves had not plateaued, suggesting incomplete diversity capture (**Figures 3C, E**). Fungal communities reached saturation (coverage >99.9%) with stabilized curves (**Figures 3D, F**). α-Diversity analysis of 50 lesional and peri-lesional samples revealed no significant differences in bacterial or fungal diversity (OTUs, ACE, Chao1, Shannon, Simpson; P>0.05, **Figures 3G–K**), though bacterial diversity significantly exceeded fungal diversity.

### 3.7 Analysis of the composition and diversity of bacterial communities

**Figure 4:**
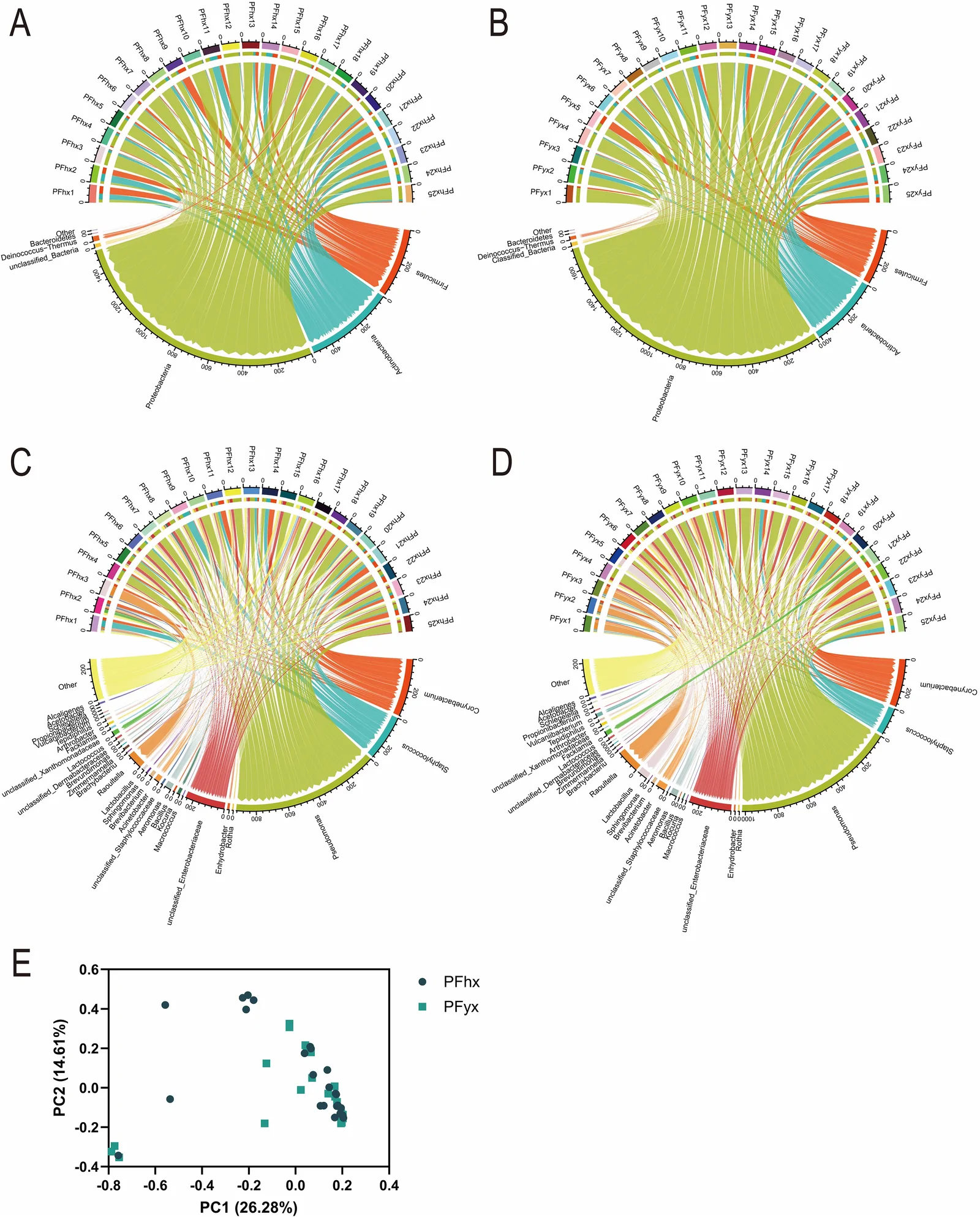
The taxonomic composition and principal component analysis (PCA) of bacterial communities in tinea corporis samples are presented. **(A)** shows the bacterial distribution at the phylum level in the skin lesion area. **(B)** illustrates the bacterial distribution at the phylum level in the surrounding skin area. **(C)** displays the bacterial distribution at the genus level in the skin lesion area. **(D)** shows the bacterial distribution at the genus level in the surrounding skin area. **(E)** presents the PCA analysis of bacteria in tinea corporis samples. PFhx indicates bacteria from the skin lesion area, PFyx indicates bacteria from the surrounding skin area.

The taxonomic classification of 2,434,411 bacterial sequences using the SILVA SSU rRNA database revealed three dominant phyla across all samples: *Proteobacteria* (64.6%), *Actinobacteria* (18.4%), and *Firmicutes* (15.2%), collectively accounting for 98.2% of total sequences (**Figures 4A, B**).

At the genus level, the 30 genera that accounted for more than 1% of all samples are shown. For bacteria in the skin lesion area (PFhx), as shown in **Figure 4C**, the top 10 most abundant bacterial genera are *Pseudomonas* (37.0%), *Corynebacterium* (14.4%), *Staphylococcus* (11.5%), *Raoultella* (4.0%), *Aeromonas* (1.7%), *Acinetobacter* (1.3%), *Facklamia* (1.1%), *Brevibacterium* (0.9%), *Kocuria* (0.8%), and *Lactobacillus* (0.6%). As shown in **Figure 4D**, the top 10 most abundant bacteria in the area surrounding the skin lesion (PFyx) are *Pseudomonas* (40.8%), *Corynebacterium* (11.9%), *Staphylococcus* (6.9%), *Raoultella* (5.8%), *Lactobacillus* (2.6%), *Acinetobacter* (2.4%), *Aeromonas* (1.7%), *Facklamia* (1.1%), *Vulcaniibacterium* (0.9%), and *Brevibacterium* (0.7%). The dominant genera in the bacterial community structure of all skin samples are *Pseudomonas*, *Corynebacterium*, *Staphylococcus*, and *Raoultella*. The differences are reflected in the higher relative abundance of *Lactobacillus* and *Vulcaniibacterium* in the surrounding area compared to the skin lesion area. PCA results (**Figure 4E**) indicate good clustering of bacteria from the skin lesion area and the surrounding area.

### 3.8 Fungal community composition and diversity analysis

**Figure 5:**
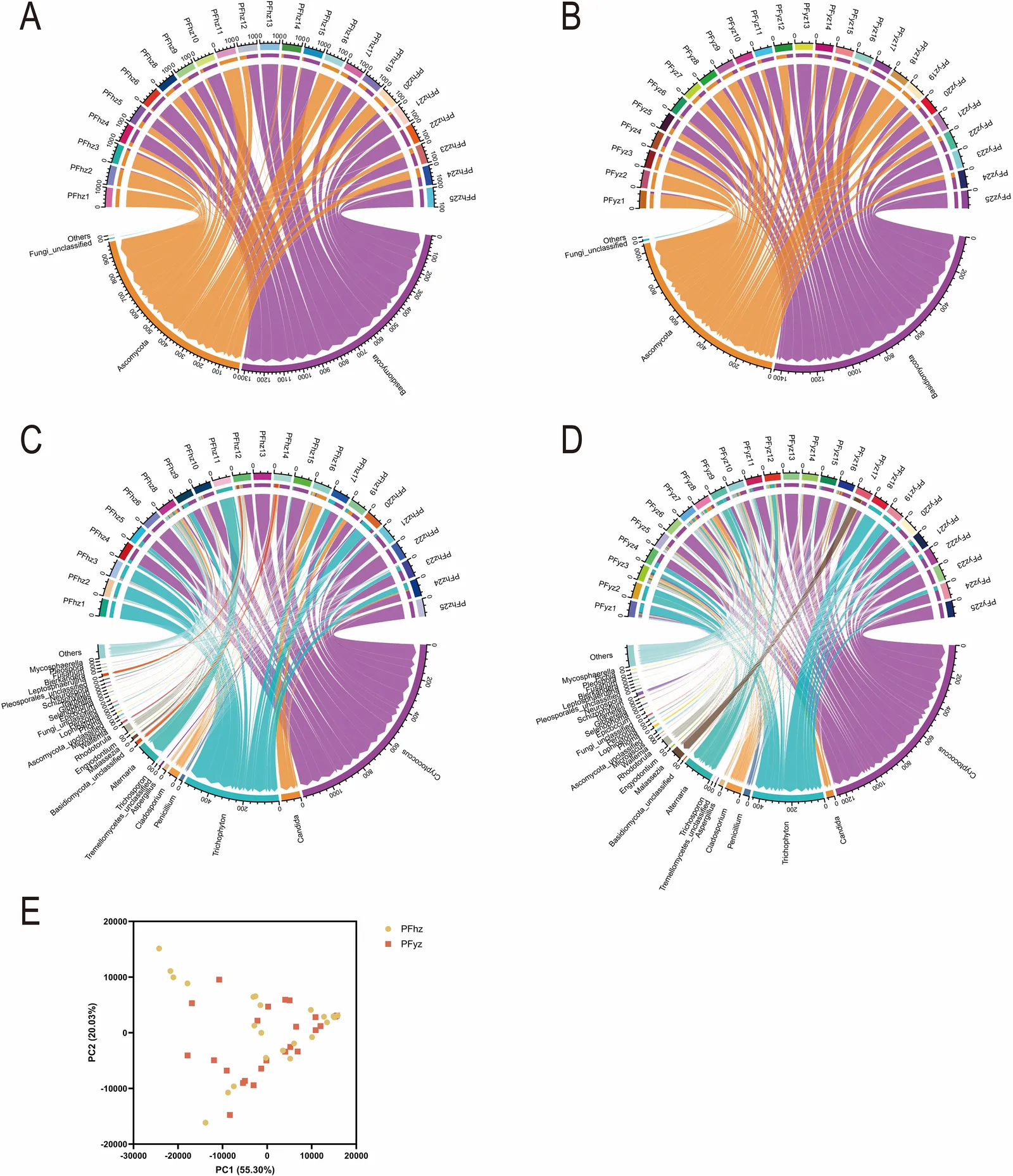
The taxonomic composition and principal component analysis (PCA) of fungal communities in tinea corporis samples are shown. **(A)** depicts the fungal distribution at the phylum level in the skin lesion area. **(B)** illustrates the fungal distribution at the phylum level in the surrounding skin area. **(C)** displays the fungal distribution at the genus level in the skin lesion area. **(D)** shows the fungal distribution at the genus level in the surrounding skin area. **(E)** presents the PCA analysis of fungi in tinea corporis samples. PFhz indicates fungi from the skin lesion area, and PFyz indicates fungi from the surrounding skin area.

The 2,434,411 filtered fungal sequences were classified using the ITS database. Fungal community structures were analyzed in both lesional and perilesional skin samples. At the phylum level (**Figures 5A, B**), the dominant fungal phyla were *Basidiomycota* (57.4%) and *Ascomycota* (42.3%).

At the genus level, 33 fungal genera with relative abundances >2% were analyzed across all samples. In lesional skin (PFhz, **Figure 5C**), the top 10 dominant fungal genera were *Cryptococcus* (51.0%), *Trichophyton* (23.7%), *Alternaria* (5.2%), *Candida* (4.7%), *Cladosporium* (2.5%), *Rhodotorula* (1.8%), *Aspergillus* (1.0%), *Penicillium* (0.9%), *Malassezia* (0.7%), and *Leptosphaerulina* (0.6%). The predominant genera in perilesional skin (PFyz, **Figure 5D**) were *Cryptococcus* (50.2%), *Trichophyton* (17.9%), *Alternaria* (7.4%), *Cladosporium* (4.0%), *Malassezia* (2.7%), *Rhodotorula* (2.0%), *Candida* (1.9%), *Penicillium* (1.6%), *Aspergillus* (1.1%), and *Neurospora* (0.8%). Cryptococcus, Trichophyton, and Alternaria dominated the core fungal community across all samples. Key differences included higher *Candida* abundance in lesional skin, while *Malassezia* and *Penicillium* were more abundant in perilesional skin. Notably, *Trichophyton rubrum* showed marked abundance variations between lesional and perilesional samples. PCA results (**Figure 5E**) revealed overlapping clustering, indicating no significant structural differences in fungal microbiota between lesional and perilesional skin.

### 3.9 Effects of culture media on *T. rubrum* growth rate and morphological characteristics

**Figure 6:**
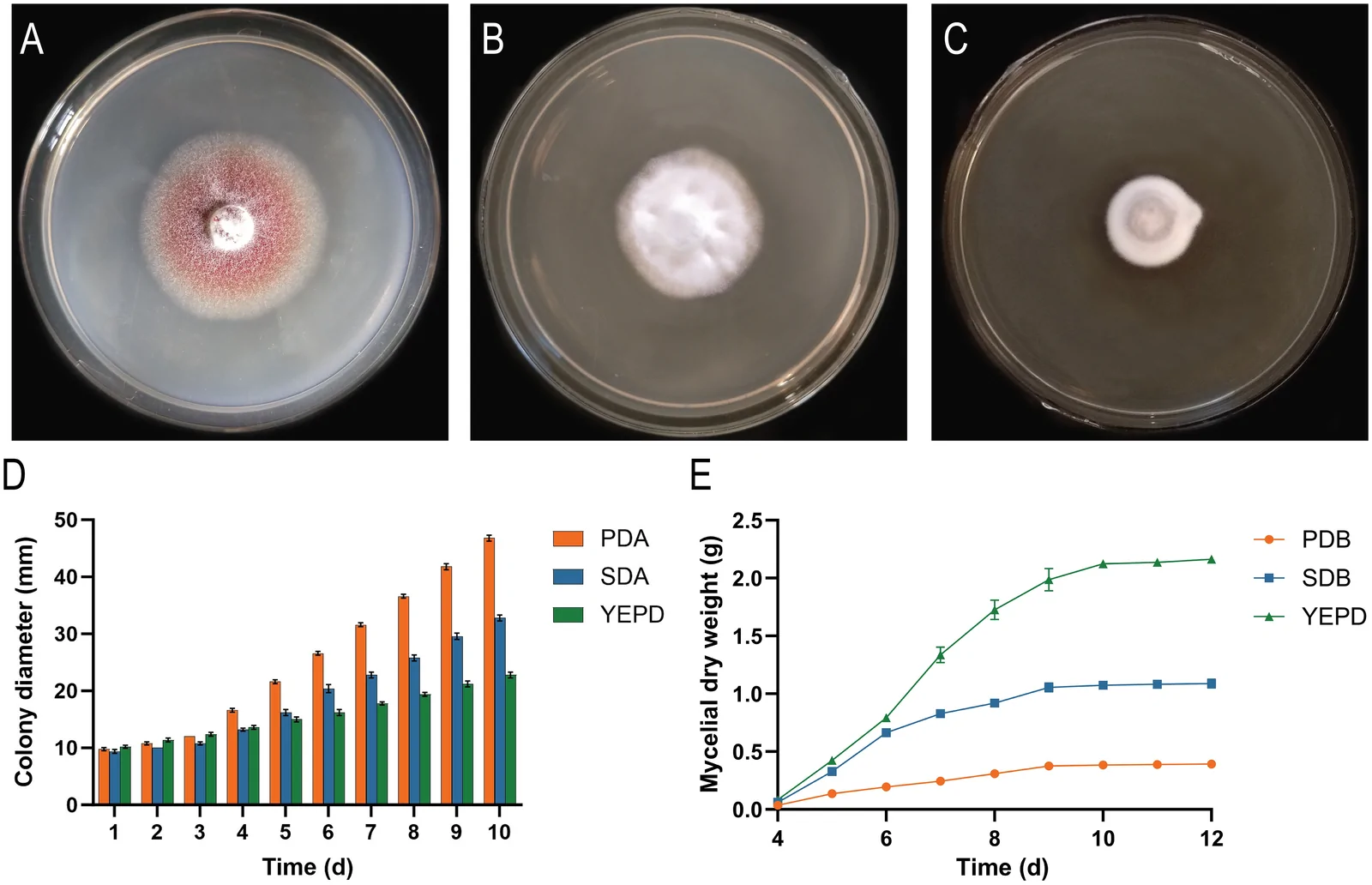
Comparison of the growth characteristics of *T. rubrum*. **(A–C)** show the morphology of *T. rubrum* on PDA, SDA, and YEPD media, respectively. **(D)** depicts the colony growth of *T. rubrum* on the three different media. E presents the mycelial growth curves of *T. rubrum* on the three different media.

*T. rubrum* exhibited distinct growth patterns across media. On solid media: PDA optimally supported colony expansion with fluffy-to-powdery textures and wine-red pigmentation (**Figure 6A**), showing the fastest growth rate. SDA yielded smaller colonies with short woolly structures and pale-yellow reverses (**Figure 6B**); YEPD induced slow growth, raised colonies, and dark-brown pigmentation (**Figure 6C**). Microscopy revealed clavate-to-pyriform microconidia and pencil-shaped, multiseptated macroconidia (**Supplementary Figure 1**). Growth rates varied significantly among solid media (**Figure 6D**), with PDA being the most favorable, followed by SDA, while YEPD performed poorly. YEPD promoted rapid mycelial biomass accumulation (dry weight) in liquid cultures (**Figure 6E**), surpassing SDB and PDB, with growth phases characterized by slow progression (days 1-4), rapid proliferation (days 4-10), and stabilization post-day 10.

### 3.10 Bacteriocin effects on biofilm formation in *T. rubrum*

**Figure 7:**
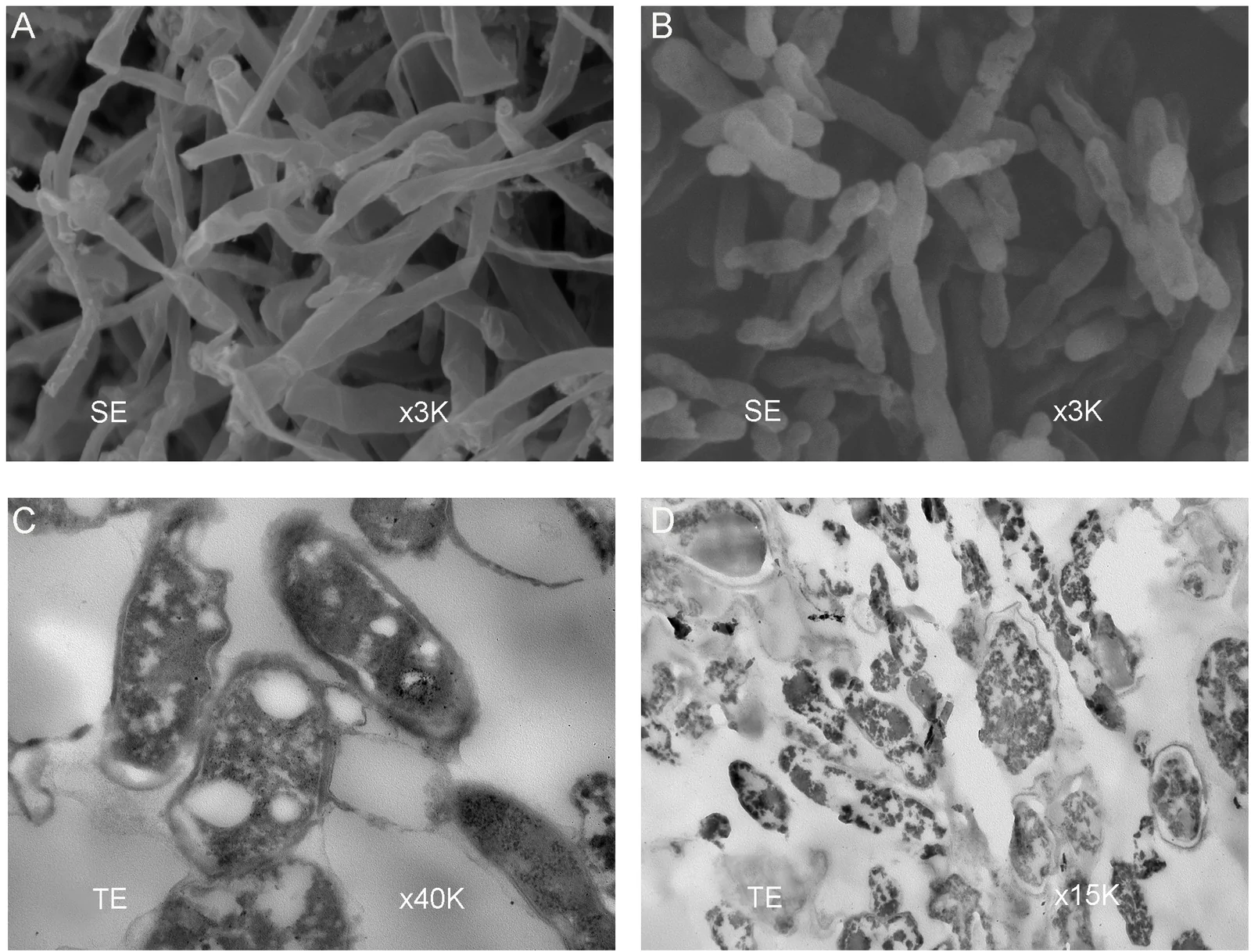
The ultrastructural changes of *T. rubrum* before and after treatment with the fermentation supernatant of the *L. plantarum* YM-4–3 strain. **(A)** shows the cell surface structure of untreated *T. rubrum* observed by SEM. **(B)** depicts the characteristics of the cell surface structure after 4 days of treatment, as observed by SEM. **(C)** illustrates the cell surface structure of untreated *T. rubrum* observed by TEM. D shows the internal cellular structure after 4 days of treatment, as observed by TEM.

Observations via SEM and TEM revealed that the antimicrobial substances produced by *L. plantarum* YM-4–3 strain significantly disrupted the cellular structure of *T. rubrum*. SEM results showed that the control group hyphae had smooth and intact surfaces with uniform structure (**Figure 7A**), while the experimental group treated with *L plantarum* YM-4–3 strain exhibited roughened surfaces, indentations, deformations, and partial fractures (**Figure 7B**). TEM further revealed that compared to the dense and regularly shaped cellular structure of the control group (**Figure 7C**), the treated group had dissolved cell walls, increased cytoplasmic transparency, and leakage of intracellular materials, along with cell aggregation (**Figure 7D**). These findings indicate that the antimicrobial substances secreted by *the L. plantarum* YM-4–3 strain can cause the death of *T. rubrum* by compromising the integrity of the cell wall and membrane permeability.

### 3.11 Inhibitory effects of L. *plantarum* YM-4–3 strain against *C. albicans* under salt stress and expression profiling of bacteriocin-related genes

**Figure 8:**
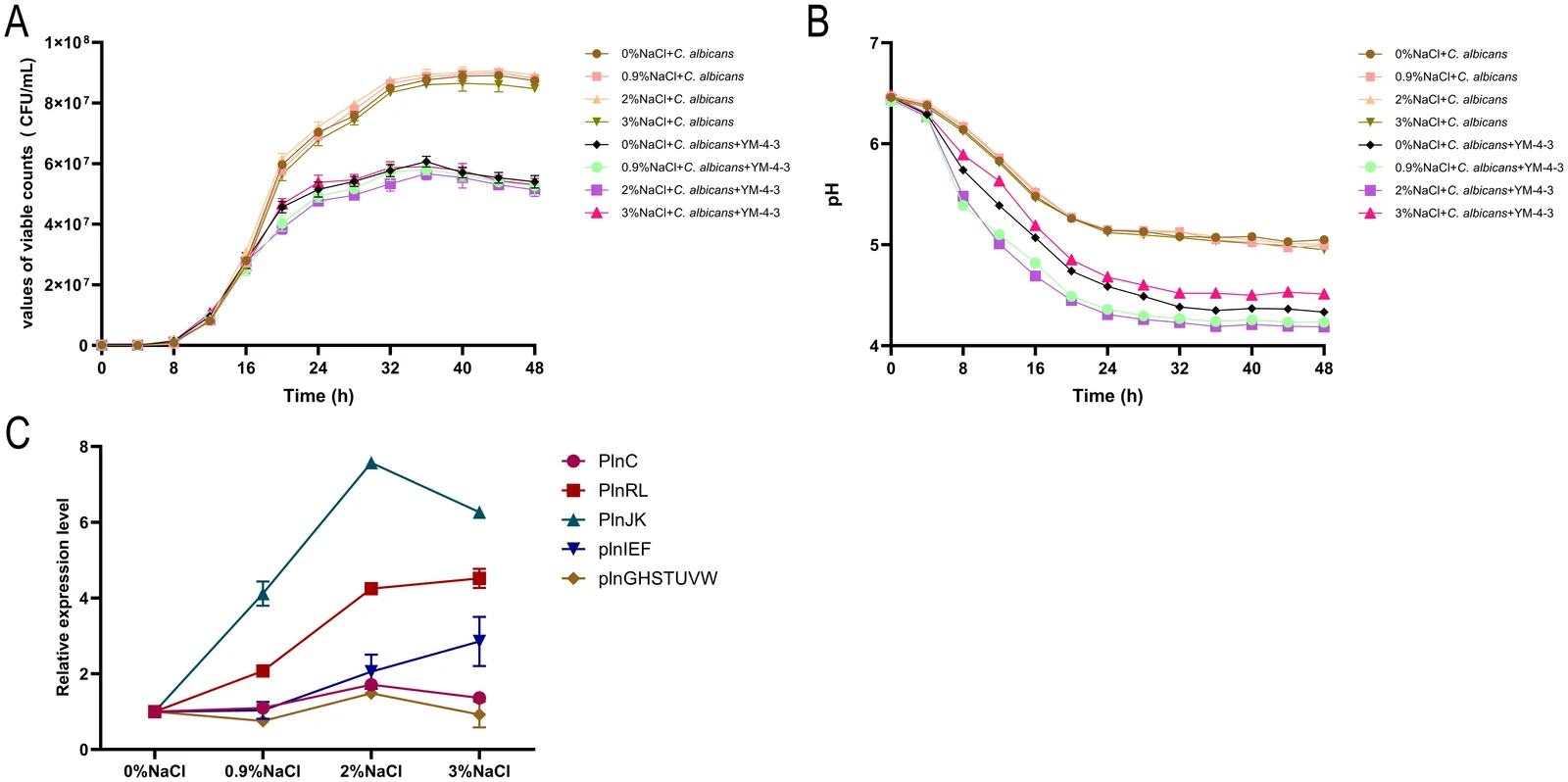
The antimicrobial activity and bacteriocin synthesis gene expression of the *L. plantarum* YM-4–3 strain under salt stress. **(A)** shows the antimicrobial effect of *L. plantarum* YM-4–3 strain on *C. albicans* under salt stress. **(B)** illustrates the pH changes of *C. albicans* strains under salt stress by *L. plantarum* YM-4–3 strain. **(C)** depicts the relative expression levels of bacteriocin genes in *L. plantarum* YM-4–3 strain under salt stress.

Co-culture experiments demonstrated that the *L. plantarum* YM-4–3 strain significantly inhibited *C. albicans* growth across varying NaCl concentrations (0.0%-3.0%). After 48 h of mixed cultivation, *C. albicans* colony counts decreased by 1.62- to 1.68-fold (p < 0.05) compared to monoculture (**Figure 8A**), confirming the strain’s antimicrobial activity. Notably, salt concentration influenced pH dynamics in the co-culture system (**Figure 8B**), with distinct gradients observed after 12 h: 2.0% NaCl < 0.9% NaCl < 0.0% NaCl < 3.0% NaCl.

qPCR analysis (**Figure 8C**) demonstrated salt concentration-dependent differential expression of bacteriocin-related genes in *L. plantarum* YM-4–3 strain during co-culture (0.0%-3.0% NaCl): *pln*IEF expression increased gradually with salinity, showing overexpression at 2% NaCl, while *pln*C exhibited an incremental trend without reaching overexpression levels, with only slightly higher expression at 2% NaCl. Notably, *pln*JK and *pln*RL were significantly upregulated within 0.9%-3.0% NaCl, with *pln*JK displaying a 7.5-fold increase at 2% NaCl compared to controls, whereas the transport system gene cluster *pln*GHSTUVW followed a similar pattern to *pln*C without overexpression, with *pln*JK and *pln*RL being the most salt-sensitive.

## 4 Discussion

This study employed high-throughput sequencing to reveal compositional differences in microbial communities between lesional and perilesional skin of patients with tinea corporis. The results demonstrated that *Proteobacteria*, *Actinobacteria*, and *Firmicutes* dominated the bacterial communities, consistent with healthy skin microbiota patterns (Ferček et al., 2025), but with significantly lower *Lactobacillus* abundance in lesional areas (0.6% vs. 2.6%), potentially reflecting its crucial role in maintaining skin homeostasis, as studies show *L. crispatus* and *L. jensenii* secrete lactic acid to maintain an acidic environment (pH<4.5) that inhibits pathogens and reduces bacterial vaginosis risk (Ravel et al., 2011), while *Propionibacterium acnes* generates short-chain fatty acids through glycerol fermentation to lower intracellular pH and suppress MRSA growth (Sanford and Gallo, 2013). Cryptococcus and Trichophyton were predominant in fungal communities, with *C. albicans* significantly more abundant in lesional areas (4.7% vs. 1.9%), possibly associated with secondary infections, collectively validating the link between skin dysbiosis and fungal infections while providing a theoretical basis for microbiota-targeted tinea corporis therapy.

During the screening of antifungal probiotics, *L. plantarum* YM-4–3 strain exhibited dual inhibitory activity against both *T. rubrum* and *C. albicans*, though its antimicrobial effects diminished with increasing salt concentrations, likely due to salt-induced suppression of bacterial growth and metabolism, reducing secretion of organic acids and antimicrobial compounds, as studies have shown salt affects bacteriocin production in *L. plantarum* (Delgado et al., 2007). Eji and Ugwuala demonstrated that while low concentrations (5%) of NaCl and KCl slightly promoted the growth of *Staphylococcus aureus*, higher concentrations (15–20%) significantly inhibited it due to osmotic stress, with NaCl showing a marginally stronger effect than KCl (Cyril Arinze and Bright Elijah, 2023). In addition, the antifungal activity of *Ruta chalepensis* essential oil also decreased under salt stress (50–100 mM NaCl) (Amdouni et al., 2016).

Electron microscopy revealed that the fermentation supernatant of the *L. plantarum* YM-4–3 disrupted *T. rubrum* cell wall integrity, inducing destructive pore formation, partial wall degradation, and cytoplasmic leakage (**Figure 7D**), similar to the mechanism of bacteriocin ZFM9 from *L. plantarum* ZFM9 against *Staphylococcus aureus* D48 (Ye et al., 2024), suggesting that the antimicrobial substances produced by *L. plantarum* YM-4–3 primarily target the cell wall and membrane of *T. rubrum*. This ultrastructural mechanism is exclusively supported by direct electron microscopy evidence from T. rubrum. For *C. albicans*, antifungal activity was confirmed via MIC assays and bacteriocin gene expression profiling, while whether a consistent ultrastructural mechanism applies to this yeast remains to be elucidated. Furthermore, the inhibitory activity of the *L. plantarum* YM-4–3 strain was found to depend on heat-sensitive substances (e.g., organic acids) and components sensitive to proteinase K. Bacteriocin-related genes (e.g., *pln*JK) showed a 7.5-fold upregulation at 2.0% NaCl, enhancing the expression of other bacteriocin genes. Studies indicate that NaCl can stimulate activity; low concentrations do not affect bacteriocin activity but may promote the expression of PBGs, thereby enhancing the antimicrobial effects of the *L. plantarum* YM-4–3 strain. In contrast, high concentrations of NaCl impair bacteriocin activity and/or reduce PBG expression, diminishing the inhibitory potential of the *L. plantarum* YM-4–3 strain (Liu et al., 2019).

Although plnJK transcription increased 7.5-fold at 2.0% NaCl, the concurrent rise in MIC indicates reduced antifungal potency. While enhanced transcription does not necessarily translate into equivalent levels of functional protein under osmotic stress, the observed reduction in activity may also be partially attributed to high salinity disrupting the electrostatic interactions between the cationic bacteriocin and the fungal cell membrane, thereby impairing membrane-targeting efficiency (Ghosh et al., 2021).

In antifungal susceptibility testing, significant variations were observed in the inhibitory effects of different antifungal agents and *L. plantarum* YM-4–3 strainfermented supernatants under varying salt concentrations against *T. rubrum* and *C. albicans*: for *T. rubrum*, ketoconazole (MIC<1 µg/mL) and terbinafine (1≤MIC ≤ 2 µg/mL) showed the strongest inhibition while 5-fluorocytosine (MIC≥64 µg/mL) was least effective, consistent with reported high efficacy of ketoconazole against dermatophytes (Adimi et al., 2013).*C. albicans* exhibited high sensitivity to amphotericin B and ketoconazole (both MIC<1 µg/mL), but resistance to fluconazole (MIC≥64 µg/mL), itraconazole (MIC>32 µg/mL), terbinafine (MIC≥64 µg/mL), and 5-fluorocytosine (MIC≥128 µg/mL), likely due to clinical overuse-induced resistance (Costa-de-Oliveira and Rodrigues, 2020). The *L. plantarum* YM-4–3 strain supernatant demonstrated notable antifungal activity against both fungi, although this activity was salt-dependent. It achieved 98% inhibition at 0.0% - 0.9% NaCl but reduced efficacy at 2.0% - 3.0% NaCl. This may be associated with salt-induced upregulation of bacteriocin genes (e.g., *pln*JK) and organic acid accumulation. These results suggest that future optimization of fermentation conditions could enhance the antimicrobial purity and activity of the *L. plantarum* YM-4–3 strain supernatant, potentially leading to the development of novel antifungal agents.

This study elucidates the microbial signatures associated with tinea corporis, demonstrates the antifungal potential of the *L. plantarum* YM-4–3 strain, and establishes a foundation for personalized therapeutic strategies combining probiotics with conventional antifungals, suggesting that targeted skin microbiota modulation may emerge as a novel paradigm for fungal infection management as microbiome research advances.

## Data Availability

The original contributions presented in the study are included in the article/**Supplementary Material**, further inquiries can be directed to the corresponding author/s.
