# Supplementary material for: Microbial community structure in tinea corporis and antifungal activity of Lactiplantibacillus plantarum YM-4–3 strain
Source: Front Cell Infect Microbiol. 2026 Aug 24;16:1839198. doi: 10.3389/fcimb.2026.1839198 (PMC13547023; doi:10.3389/fcimb.2026.1839198)
Supplement: Supplementary file 1 [file Supplementaryfile1.docx]

Fig. S1 Hyphal and conidial structures of *Trichophyton rubrum* observed under microscope. Hyphae (yellow arrows), clavate-to-pyriform microconidia (black arrows), and pencil-shaped, multiseptated macroconidia (white arrows) are visible. Scale bar, 25 µm.
